# ASO Author Reflections: Patterns, Timing, and Survival of Recurrence after Surgery for Esophageal and Junctional Adenocarcinoma

**DOI:** 10.1245/s10434-026-19462-y

**Published:** 2026-03-17

**Authors:** Lucas Goense, Jessie A. Elliott, Richard van Hillegersberg, John V. Reynolds

**Affiliations:** 1 Department of Surgery, University Medical Center Utrecht, Utrecht University, Utrecht, The Netherlands; 2 Department of Surgery, Trinity St. James’s Cancer Institute, Trinity College Dublin, St. James’s Hospital, Dublin, Ireland

## Past

Recurrence after curative intent treatment of esophageal adenocarcinoma is common and remains the main driver of survival. Historically, recurrence has often been simplified into locoregional versus distant categories, with distant metastases generally assumed to carry uniformly poor survival outcomes.^1^ However, clinical experience indicates substantial heterogeneity. Some patients relapse early with rapidly progressive systemic disease, whereas others develop later recurrences with limited disease that may be considered for tumor-directed therapy. Therefore, important questions remain regarding the incidence of specific recurrence sites, differences in time to recurrence, and whether these variations are associated with distinct prognostic and therapeutic implications.

## Present

The analysis of recurrence patterns within the ENSURE study demonstrates that recurrence is not a single entity.^2,3^ Liver-only and multisite recurrences occur earliest and are associated with the poorest post-recurrence survival. In contrast, isolated locoregional and lung-only recurrences occur later and are associated with substantially longer survival after detection. The recurrence-free interval itself is a strong prognostic marker, with later recurrence translating into better outcomes than those with early relapse. These findings have direct clinical implications. Early liver or multisite recurrence should primarily prompt systemic therapy and clinical trial consideration. In this context, systemic treatment was associated with longer post-recurrence survival compared with best supportive care. Conversely, selected patients with late, limited locoregional or pulmonary recurrence may benefit from aggressive local, tumor-directed treatment. Recurrence site and timing therefore provide readily available surrogates of tumor biology that can guide decision-making beyond a uniform palliative approach.

## Future

Recurrence pattern and recurrence-free interval should be integrated in treatment decision-making while explicitly accounting for patient fitness and performance status. Prospective trials and registries such as OMEC are on their way to defining which patients with recurrence may be candidates for metastasis-directed surgery or radiotherapy alongside available systemic and immunotherapeutic regimens.^4^ The integration of molecular profiling and liquid biopsy techniques in post-treatment surveillance will enable identification of molecular relapse before radiologically overt, widespread disease develops, and may facilitate targeted treatment. This could create a window for earlier intervention in high-risk patients, with the aim of delaying or preventing progression to clinically evident systemic disease. Such individualized, biologically informed strategies including novel immunotherapy approaches offer the most promising route to improving outcomes for patients with esophageal cancer into the future.
